# Biomechanics of Neutrophil Tethers

**DOI:** 10.3390/life11060515

**Published:** 2021-05-31

**Authors:** Andrea Cugno, Alex Marki, Klaus Ley

**Affiliations:** 1 La Jolla Institute for Allergy and Immunology, La Jolla, CA 92037, USA; acugno@lji.org (A.C.); amarki@lji.org (A.M.); 2 Department of Bioengineering, University of California San Diego, La Jolla, CA 92093, USA

**Keywords:** tether breakage, ENDS formation, tether pulling, nonlinearly decaying springs, cell mechanics, viscoelasticity, mathematical modeling, mechanobiology

## Abstract

Leukocytes, including neutrophils, propelled by blood flow, can roll on inflamed endothelium using transient bonds between selectins and their ligands, and integrins and their ligands. When such receptor–ligand bonds last long enough, the leukocyte microvilli become extended and eventually form thin, 20 µm long tethers. Tether formation can be observed in blood vessels in vivo and in microfluidic flow chambers. Tethers can also be extracted using micropipette aspiration, biomembrane force probe, optical trap, or atomic force microscopy approaches. Here, we review the biomechanical properties of leukocyte tethers as gleaned from such measurements and discuss the advantages and disadvantages of each approach. We also review and discuss viscoelastic models that describe the dependence of tether formation on time, force, rate of loading, and cell activation. We close by emphasizing the need to combine experimental observations with quantitative models and computer simulations to understand how tether formation is affected by membrane tension, membrane reservoir, and interactions of the membrane with the cytoskeleton.

## 1. Introduction

Neutrophils, or polymorphonuclear (PMN) leukocytes, have a crucial role in the host response to bacterial, fungal, and viral infections [1,2]. Neutrophils also have roles in several chronic diseases, such as atherosclerosis, cancer, allergy, and autoimmune diseases [3]. In adult mammals, neutrophils are primarily made in the bone marrow, from where they are released into the blood circulation. The circulating neutrophils are recruited into the tissue through a cascade of events consisting of rolling on the vessel wall, arrest, crawling, and transmigration. During transmigration, the neutrophils leave the vessel lumen toward the inflamed tissue following chemotactic clues [1,4].

**Figure 1:**
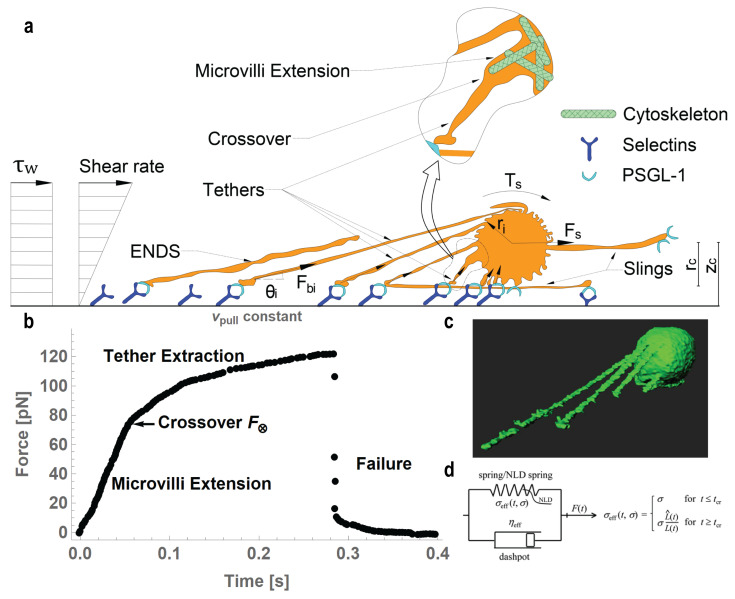
Schematic of rolling neutrophils in a Couette flow (**a**): in the zooming inset, the tether formation from an extended microvillus. (**b**) Typical mechanical record of a tether pulling experiment at a constant pulling speed $v_{pull}$ (reproduced for illustrative purposes from [49]). (**c**) Three-dimensional reconstruction image of an arrested neutrophil from [5] with permission, showing multiple tethers extracted. (**d**) Schematic of the nonlinearly decaying spring viscoelastic model capturing the complete behavior during tether extraction; the elastic component has two different behaviors before and after crossover.

In vitro and in vivo studies have shown that, to reach areas of inflammation, PMNs first are captured and then roll along the vascular endothelium under wall shear stress <40 dyn/cm${}^{2}$ [3,4,5,6,7]. During rolling, neutrophils come into close contact with the chemokines presented on the endothelial surface, which activates integrins and triggers arrest. The clues for crawling on the luminal side of the endothelium and for transmigration are less clear [1,4,8,9,10,11]. Rolling is mediated by a series of molecular bonds between receptors on the surface of neutrophils and ligands on endothelial cells (EC) that rapidly form and dissociate [4,12,13,14,15,16]. These molecular bonds are mediated by the selectin family of adhesion molecules, P-, E-, and L-selectin [17,18,19,20,21,22,23,24]. Throughout this review, we refer to “bond” as the sum of noncovalent interactions, such as hydrogen bonds, electrostatic interactions, van der Waals forces, and dipole–dipole interactions, between two or more macromolecules [22]. With the onset of inflammation, the venular endothelial cells rapidly expose P-selectin on their surface by fusing their P-selectin-containing storage granules (Weibel–Palade bodies) to their luminal plasma membrane. As the neutrophils squeeze through the capillaries and enter the venules, they come into contact with the P-selectin surface [25]. Through binding with P-selectin glycoprotein ligand-1 (PSGL-1), they begin rolling on the venular endothelium at velocities between 50 and 200 µm/s [18,26]. Neutrophils reduce their rolling speed (<10 µm/s) as their β2-integrins become activated by PSGL-1-induced signaling and bind to intercellular adhesion molecules (ICAMs) on the endothelial surface [27]. About two hours after the onset of inflammation, the endothelial cells begin to present E-selectin on their surface, which enables “slow” rolling at speeds less than ∼10 µm/s [26] by binding to PSGL-1, ESL-1, and CD44 on the neutrophil surface [28]. Rolling is not always necessary for neutrophil recruitment, but it is crucial in host defense, as shown by recurrent severe infections in patients with defective selectin ligands [29,30]. Neutrophils have excess membrane folded into cytoskeleton-linked surfaces ruffles, called microvilli [31,32], where PSGL-1 is concentrated and initiates contact with the vessel wall [7,33]. The bonds between selectins and PSGL-1 have a characteristic force-dependent behavior [24]. Depending on the force acting on the bond, the receptor–ligand bond’s lifetime may decrease (slip bond) or increase (catch bond) [24]. The bonds between microvilli and ECs work against the shear forces and torques exerted on the leukocyte by the flowing blood. Circulating neutrophils in postcapillary venules move at >1000 µm/s transported by blood flow [25], but during rolling after initial contact with the vessel wall, they sustain a sharp reduction in speed down to 100, 20, or even 2 µm/s. The force exerted at the location of the bond can extend the microvilli into cell protrusions (Figure 1), and if the force exceeds a threshold ($F_{th}≃35$ pN), the plasma membrane can separate from the underlying cytoskeleton and a membrane tether can be formed (Figure 1a) [16,33,34]. Tethers are ∼200 nm thin and up to ∼20 µm long, approximately cylindrical structures that extend from the surface of the microvilli [5,11,35,36]. Adherent neutrophils in vitro form cytonemes that are of similar diameter and length as force-induced tethers [37,38]. Cytonemes likely develop from frustrated secretion and contain bactericidal peptides [39]. Nitric oxide [40,41] and cytochalsin D [39,42] can induce cytonemes. Unlike tethers, cytonemes form in the absence of flow and take minutes, not seconds, to form [37,38,40,43].

**Figure 2:**
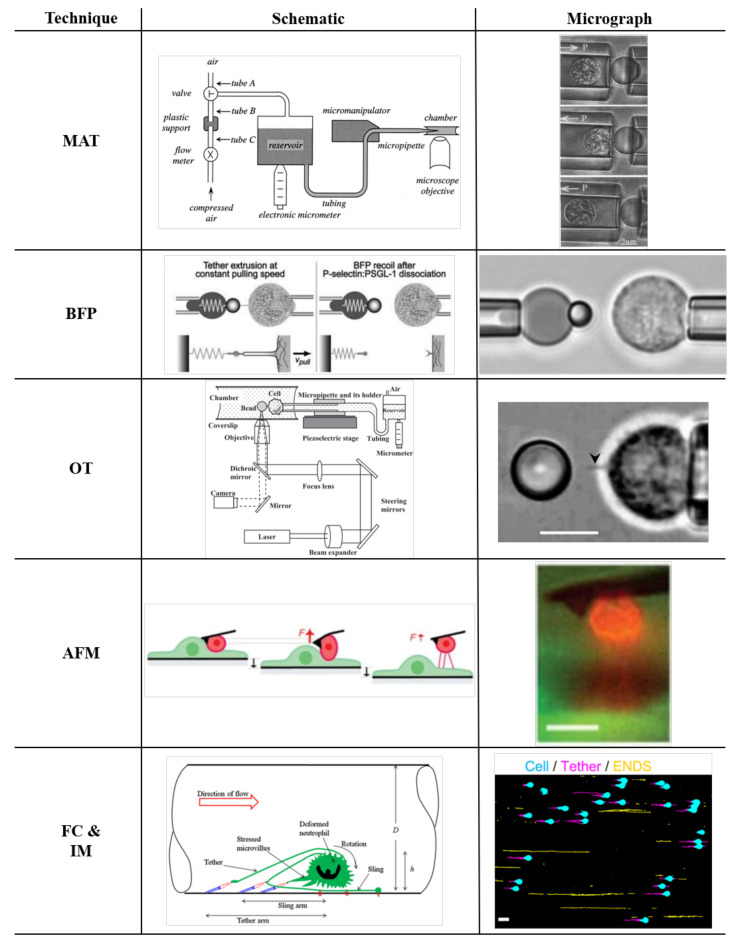
Experimental techniques used for tether extraction. For each method, the **Schematic** (SC column) and the **Micrograph** (MG column) are reported. Micropipette aspiration (**MAT** row), SC with permission from [50], and MG with permission from [44]; biomembrane force probe (**BFP** row) SC and MG with permission from [52]; optical trap (**OT** row) SC and MG with permission from [56]; atomic force microscopy (**AFM** row) SC and MG with permission from [58]; and flow chamber and intravital microscopy (**FC and IM** row) SC with permission from [48] and MG with permission from [35].

The neutrophils’ ability to extend microvilli and to form tethers under a pulling force was initially shown by Shao and Hochmuth [44] with micropipette aspiration (Figure 2). During their experiments, a neutrophil held in a suction pipette was brought into contact with a bead coated with adhesion molecules. The neutrophil was moved away from the bead by applying suction, and the neutrophil displacement under different forces was measured by brightfield microscopy. These experiments indicated that a tether must be present between the neutrophils and the beads, but the tethers were not visible directly. Since the tethers’ diameter is smaller than the resolution of diffraction-limited optical microscopy, imaging of tethers with brightfield microscopy requires contrast enhancement techniques. Tethers of rolling neutrophils were first visualized in a flow chamber with differential interference contrast (DIC) microscopy [11,36,45]. Later, fluorescent plasma membrane labeling techniques were adopted that enabled detailed neutrophil tether imaging in flow chambers and in living mice [5,7,35,46]. The labeling techniques allowed us to analyze tether formation from rolling neutrophils in mouse blood vessels or in vitro reproduced physiological flow conditions. These studies confirmed that tethers are load-bearing structures because a sudden increase in rolling velocity was observed after their breakage [5,7,35,46]. From the biomechanical point of view, both microvillus extension and tether formation are fundamental mechanisms to increase the lifetime of molecular bonds because they can decrease the pulling force imposed on the adhesive bonds at high shear stresses [34,47,48]. Tethers can break at the anchor point, where they are attached to the adhesive substrate, resulting in a sudden transitory increase of the rolling speed (jump) [5,6,7,35,46]. Tethers can also break between the anchor point and the neutrophil cell body (see Figure 1 and micrograph in Figure 2 FC and IM row), resulting in left-behind pieces of neutrophils called elongated neutrophil-derived structures (ENDS) [35]. While these imaging experiments showed the tethers© life cycle, they were not suited for biomechanical characterization of tether formation and failure.

Various techniques have been used to characterize tether pulling from neutrophils. The Micropipette Aspiration Technique (MAT) [34,44,50,51] and Biomembrane Force Probe (BFP) [49,52,53,54] take advantage of a micropipette manipulation system with two opposing coaxial pipettes, one holding the force transducer (a cell or a protein-coated bead) and the other holding the object of interest (Figure 2). In an Optical Trap (OT) [55,56] setup, one of the coaxial pipettes is replaced with a system producing trapping forces by applying a laser beam to a coated microbead. The cell is moved through a micropipette manipulation system, which imposes displacement, and detecting the bead’s deflection makes it possible to measure the forces needed to keep the bead trapped. Atomic force microscopy (AFM) uses a sharp tip that allows to impose or measure forces with nanometer spatial resolution and sensitivity of the order of tens of piconewtons [55,57,58,59,60]. All of these techniques were also employed to pull tethers (or nanotubes) from lipid vesicles, which are the simplest model to approximate cellular membrane properties [61]. One of the advantages of using lipid vesicles is that it is possible to control their membrane composition and to create special packaging allowing for the presence of an internal actin shell. With membrane vesicles, it is possible to isolate the effects of the membrane versus the shell, thus providing crucial insights on how the membrane composition [62,63,64], the presence of a cytoskeleton, and the crosslinking proteins between the plasma membrane and cytoskeleton affect tether extraction [61,65,66,67,68]. Actin filaments have been observed inside tethers when extracted from various cells even after treatment with cytochalasin D, a drug that disrupts actin filaments [69,70,71]. The presence of actin filaments inside neutrophil tethers has not been demonstrated yet. If neutrophil tethers contain actin filaments, this will affect tether biomechanics, tether extraction, and tether breakage. The potential presence of actin filaments within neutrophil tethers inevitably would reinforce the membrane. If F-actin filaments along tethers were nonuniform, this could contribute to the formation of weak spots where the tether could break. Indeed, tethers have been observed to break at any point between the tether anchor point, where the tether is attached to the substrate, and the tether neck, where the tether emanates from the body of the neutrophil thus forming ENDS [35]. Membrane mechanics computational simulations suggest that the concentration of tangential stress in the neck area, where tethers emerge from the cell, could cause the formation of such weak spots [72].

Many phenomenological mechanical relations for the dynamical behavior of tether extraction have been provided based on agreement with at least one experiment [24]. The dynamic nature of cells, biological tissues, and tether extraction experiments is often described with viscoelastic models [16,73,74,75,76]. Pospieszalska et al. [77] introduced a viscoelastic model incorporating a nonlinear decaying spring (NLD), providing a unified approach to describe and replicate published protrusion and tether pulling experiments with a single model. This model was also used to estimate the force exerted on tethers during in vivo and in vitro flow experiments [7,24,48].

This review provides an overview of the current understanding of the biomechanics of neutrophil tether formation. We start by reviewing the fundamental physics behind tether formation. We discuss the different techniques used to pull tethers and to observe neutrophil tether formation during in vivo or in vitro rolling experiments. We also report insights from nanotubes pulled from giant unilamellar vesicles (GUVs) and finally discuss future directions that could move the field forward.

## 2. Fundamental Physics of Tether Formation

Neutrophils roll along the blood vessel wall with a characteristic jerky-tumbling motion [12,23,24,26] propelled by the shearing forces of the blood on their lumen-facing side and by hydrostatic pressure on their upstream-facing side, and modify the shear field experienced by the vessel wall when rolling and tethering [14,78]. The shear flow induces a shear force $F_{s}$ along the flow direction and a shear torque $T_{s}$ around the center of the rolling neutrophils (Figure 1a). Estimations of the force $F_{s}$ and torque $T_{s}$ for a stationary sphere of radius $r_{c}$ in a Couette flow, under the assumption of very small Reynolds numbers (≪1), have been provided by Goldman’s expressions [79]. Solving for the shear force $F_{s}$ and torque $T_{s}$, we have

(1) $$F_{s}=6\pi F_{s}^{*}r_{c}z_{c}\tau_{w},\;\mathrm{and}$$

(2) $$T_{s}=4\pi T_{s}^{*}r_{c}^{3}\tau_{w}.$$

Equations (1) and (2) are valid when the ratio $\frac{z_{c}}{r_{c}}\geq 1$, where $r_{c}$ is the radius of the cell. $F_{s}^{*}$ and $T_{s}^{*}$ are numerical parameters depending on the ratio $\frac{z_{c}}{r_{c}}$ ($z_{c}$ is the cell’s center distance from the substrate), and $\tau_{w}$ is the wall shear stress [79].

Selectin-mediated molecular bonds between the tips of the microvilli (∼200 nm high cell protrusions [80]) slow down the rolling cell. The bonds’ forces ($F_{b}$) serve as an automatic braking system [81,82] slowing down or stopping the rolling if the forces are large enough to balance the shear force and torque. The dynamic equilibrium of forces in the tangential (→) and normal (↑) directions to the flow, and the torque about the center of the rolling neutrophils (⭮) are given, respectively, by the following (Figure 1a):

(3) $$\to :\;F_{s}=\sum_{i=1}^{N}F_{bi}\;cos\theta_{i},$$

(4) $$↑:\;F_{N}=\sum_{i=1}^{N}F_{bi}\;sin\theta_{i},$$

(5) $$⭮:\;T_{s}=\sum_{i=1}^{N}F_{bi}\times r_{i}.$$

In Equations (3)–(5), *N* is the number of active bonds (bound to ligand), $\theta_{i}$ is the angle between the *i*th bond force vector and the flow direction (Figure 1a), and $F_{n}$ is the force normal to the substrate (“downforce”). The first term on the right-hand side of Equation (5) is the sum of all torques generated at the surface of the neutrophil by all bond force vectors; the symbol “×” represents the vector product; and $r_{i}$ is the position vector of the neck of the protrusion, where the force is transferred to the cell’s surface. We neglect inertia because, in venules and flow chambers, Reynold’s number is sufficiently small (≪1).

Quantitative dynamic footprinting (qDF) microscopy combined with modeling [6,7,16] revealed that, when neutrophils roll, the bonds form in the front of the cells; are compressed under the center; and are in tension at the rear of the footprint, where they finally break away from the substrate [7] (Figure 1a). Neutrophils have a remarkable ability to form surface protrusions and tethers in response to a pulling force $F_{b}$ applied locally to the cell membrane (Figure 1). Surface protrusions are viscoelastic tubular structures formed under low pulling forces, whereas tethers are thin tubes formed under larger forces. If an initially low pulling force starts increasing (Figure 1), first a protrusion and then a tether from the extended microvillus are formed. From the biomechanical point of view, both microvillus extension and tether formation are essential mechanism that increase the lifetime of the molecular bonds because they decrease the pulling force imposed on the adhesive bonds at high physiological shear stresses [34,47]. In vitro and in vivo cellular protrusions and tethers, pulled using molecular bonds, were studied in flow chamber [5,7], intravital microscopy [35,46], MAT [44,50], BFP [49,52], OT [56], and AFM experiments [58,83,84].

The two phases of the process, from protrusion formation to tether extraction, are characterized by two different material dynamical behaviors, as shown by the time course of the force during BFP pulling experiments at a constant velocity and reproduced here from [49] for illustrative purposes (see Figure 1b). Initially, the dynamic behavior of the microvillus extension is viscoelastic. The viscous character comes from the lipid membrane flowing into the tether, whereas the elastic behavior comes from the cytoskeletal elements in the tether stretching such as a spring [16,48,85,86]. The transition between microvilli extension to tether extraction is called a crossover. Crossover is believed to occur when the plasma membrane detaches from the underlying cytoskeleton and starts flowing around transmembrane proteins bound to the cytoskeleton and into the tether [87,88,89]. During the second phase, the tether’s elastic property diminishes over time as the pulling force increases. Experiments performed with MAT [44] showed a viscous behavior, likely because, with this technique, the initial viscoelastic phase was not resolved. Tethers extracted with OT, BFP, and AFM [49,56,85] were all modeled with viscoelastic models. Those experiments also indicated that the crossover event and the pulling force at crossover depends not only on the cell but also on the pulling method and pulling speed. Indeed, a theoretical framework for tether formation [86,87,88,89] proposed that the threshold force $F_{th}$, the smallest force above which the crossover can occur, can be obtained from the mechanical energy (*U*) of a membrane tether of length *L* and radius $r_{t}$, such as

(6) $$U=\frac{\pi \kappa }{\;r_{t}}L+2\pi r_{t}\left(\sigma_{m}+W_{0}\right)L,$$

where κ is the membrane bending modulus, $\sigma_{m}$ is the membrane tension that arises from the pressure difference across the bilayer, and $W_{0}$ is the adhesion energy (per unit area of the membrane) to the cytoskeleton arising from the pulling force from the cortex and the cytoskeleton filaments [69,86,90]. The adhesion energy is a measure of the *glue* that binds together the plasma membrane and the underlying cortical cytoskeleton. Its molecular mechanism involves a layer of specialized crosslinking proteins (such as ezrin, radixin, and moesin) capable of binding to lipids in the membrane (e.g., PIP2) and F-actin. It also involves nonspecific frictional forces between the cortex and the membrane (i.e., electrostatic or van der Waals forces) [59,91,92,93,94].

Subsequently, choosing as independent kinematic parameters in Equation (6), the length (*L*), and the volume of the tether ($\Omega =\pi r_{t}^{2}L$), the incremental work of extrusion of the tether is

(7) $$F_{th}dL=dU+pd\Omega ,$$

where *p* is the hydrostatic pressure inside the tether. Thus, $F_{th}$ is equivalent to the static force needed to pull a tether given by

(8) $$F_{th}=\frac{\pi \kappa }{\;r_{t}}+2\pi r_{t}T_{m},$$

with $T_{m}=\sigma_{m}+W_{0}$ being the total *effective membrane tension*. It is worth highlighting that the membrane tension $\sigma_{m}$, the measure of the energetic cost of increasing the membrane area (measured in $J/m^{2}=N/m$ ), can be affected by the pulling method and can thus alter the measured tether force. As an example, when cells of radius $r_{c}$ are sucked through micropipettes of radius $R_{p}$ with a pressure Δp during MAT, OT, and BFP pulling experiments, the membrane tension is set by the Laplace law as follows [59,61,65,91,95],

(9) $$\sigma_{m}=\Delta p\frac{R_{p}r_{c}}{2\left(r_{c}-R_{p}\right)}.$$

During a dynamic tether pulling, if the force exceeds $F_{th}$, tethers can undergo a crossover, where the membrane starts to flow into the tether from a cell membrane reservoir [69,70,96]. The tether force and the elongation rate $\dot{L}$ are set by the following equilibrium formula [86,87,88,89],

(10) $$\dot{L}=\left[F^{3}-FF_{th}^{2}\right]/\left[16\pi^{3}\kappa^{2}b_{eff}ln\left(r_{c}F/2\pi \kappa \right)\right],$$

with parameters defined as in Equation (4) and with $b_{eff}$ being the cell membrane interfacial drag coefficient (i.e., the surface density of the bound transmembrane proteins multiplied by the surface viscosity of the plasma membrane). The crossover force $F_{⊗}$ is the value of the force when Equation (10) becomes valid (Figure 1b). Dynamic microvilli extension and tether extraction represent two moments with two different material behaviors of the same phenomenon. Pospieszalska and Ley [77] introduced a unifying model, making use of nonlinearly decaying spring (NLDs) viscoelastic material (Figure 1d); derived a methodology to estimate time and force at crossover ($t_{⊗}$ and $F_{⊗}$); and characterized both phases during tether extraction experiments. Importantly, crossover occurs if a dynamic equilibrium is reached and can occur (necessary but not sufficient condition) if the force is higher than the threshold force $F_{th}$. Before crossover ($t<t_{⊗}$), the tether can be represented with a classical Kelvin–Voigt viscoelastic model consisting of a viscous element (dashpot) in parallel with an elastic element (spring) for which the pulling force on the tether is

(11) $$F\left(t\right)=kL\left(t\right)+\eta_{eff}\dot{L}\left(t\right),$$

where *k* is the tether spring constant and $\eta_{eff}$ is the effective tether viscosity. After crossover, tethers start to flow following the equilibrium formula in Equation (10), gradually losing their elastic component. The model reproduced with high precision the experiments reported in Shao et al. [47], Xu and Shao [56], Evans et al. [49], Heinrich et al. [52], and provided estimations of the tether force during flow chamber experiments [7,24,48].

Equations (10) and (11) show an essential feature of tethers: the force is positively correlated with tether elongation rate. As neutrophils roll in the blood vessel, the selectin bonds extract multiple tethers that reduce the rolling velocity (and the elongation rate), effectively reducing the force on each tether, hence reducing the force on the bonds and increasing their lifetime, which stabilizes the rolling [31,47]. Furthermore, the tethers’ diminished ability to retract at higher pulling forces (decaying spring) is thought to contribute to the formation of slings [7,48]. When the long tether detaches from the substrate do not retract quickly but rather rotate with the rotating cell, giving rise to slings. Slings are another mechanism that stabilizes rolling because they roll with the neutrophils and appear at the cell’s front. On slings, PSGL-1 is organized in patches that can reform molecular bonds with L-selectin that become loaded again with the cell’s rolling. The tethers’ ability to retract is not entirely lost and even long tethers retract given sufficient time [35]. Recent intravital imaging showed that tethers can also break between the anchor point and the cells, resulting in left-behind elongated pieces of neutrophils (ENDS), which recoil into a spherical shape (energetically most favorable) in about 4 h [35].

## 3. Experimental Methods to Pull Tethers

### 3.1. Tether Extraction with Micropipette Aspiration Technique

Micropipette aspiration (MAT) is often used to study the mechanical behavior of living cells [97], and in particular, it is a suitable technique to measure the overall cellular tension [59]. The first investigation of tether formation using a micropipette manipulation system was performed in RBCs. The technique was slightly modified using a micropipette manipulation system with two opposing coaxial pipettes [33,34,44,85,98,99]. One of the pipettes holds protein-coated beads, and the other holds the cell of interest (Figure 2, MAT row). The technique was used to pull tethers from different cells. Several studies showed tether formation in neutrophils using beads coated with antibodies against various cell surface proteins, the transmembrane phosphatase CD45, the β2 integrin CD18, L-selectin (CD62L), PSGL-1 (CD162), or the hyaluronan receptor CD44. Antibodies have high affinities and low off-rates, thus ensuring strong and lasting molecular bonds. A pump actuates the force transducer (the cell or the protein-coated bead [50]), allowing for contact between the bead and the cell first and then extracting the tether through suction (see Figure 2). The force imposed by the pipette directly depends on the hydrostatic pressure by the relation

(12) $$F_{MAT}=\pi R_{p}^{2}\Delta p,$$

where $R_{p}$ is the radius of the moving particle and Δp is the hydrostatic pressure imposed [44]. When the tethers form, the actuated particle’s velocity is lower than when the same particle when is free, and the tether force is calculated from their difference, as shown by the following relation:

(13) $$F_{t}=F_{MAT}\left(1-\frac{4}{3}\frac{R_{p}-R_{m}}{R_{p}}\right)\left(1-\frac{v_{pull}}{v_{f}}\right),$$

where $R_{m}$ is the radius of the micropipette and where $v_{pull}$ and $v_{f}$ are the velocity of the tethered and freely moving cell, respectively. No calibration is required because the tether force (Equation (13)) is derived from the Naiver–Stokes equation’s solution for a sphere moving inside a cylindrical tube containing a viscous medium, under the hypothesis that the sphere moves at a constant velocity [44]. Perfect sealing between the particle and the pipette is not required ($R_{p}-R_{m}\geq 0$), but the precision of Equation (13) increases when the relative difference between the radius of the pipette and the sphere is small ($\left(R_{p}-R_{m}\right)/R_{m}=\varepsilon \to 0$). The microscope measures the particle’s speed, thus giving an indirect measure of the force on the tether through Equation (13). The method allows us to measure the tether pulling forces with piconewton sensitivity, ranging between 0 to 300 pN [44,50,100] depending on the pulling velocity, with sub ms time resolution, since this can be performed by measuring the distance between the neutrophil and beads on brightfield images.

**Figure 3:**
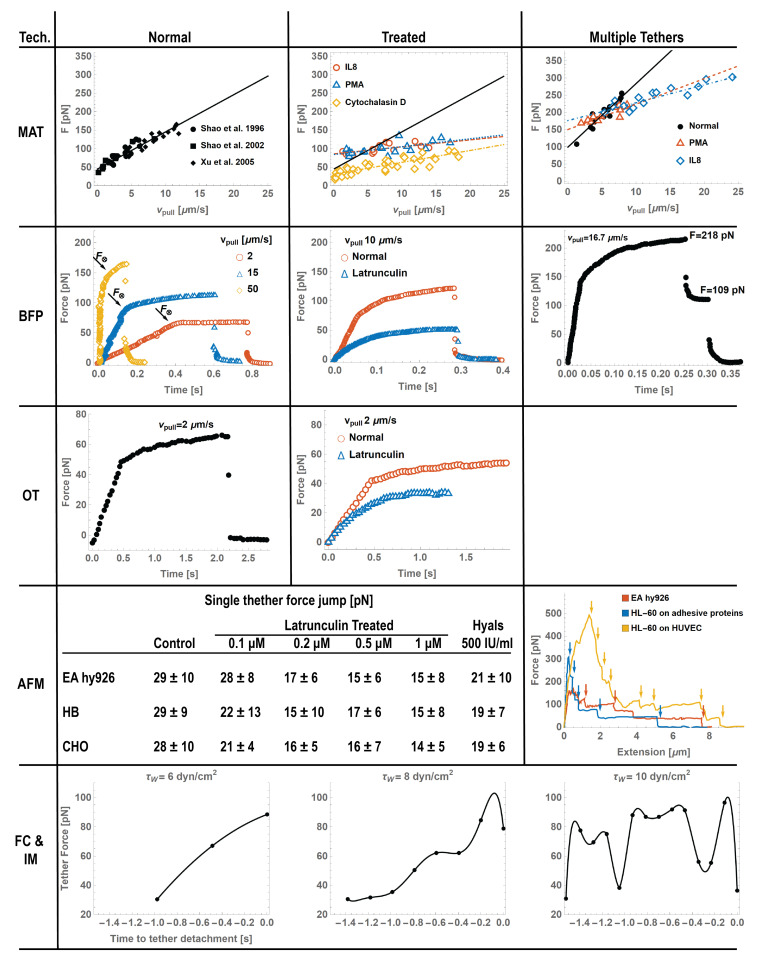
Force measurements with different techniques are reported here for illustrative purposes. For each technique, we showed the behavior of single **Normal** (N column), **reated** (T column), and **Multiple** (M column) tethers when present. **MAT** row from [44,50,100]. The solid line in N and the dashed one in T columns show the linear correlation ($F_{th}=45$ pN and $\eta_{eff}=1.8$ pN s/µm in Equation (14)) between the tether growth velocity $v_{pull}$ and the force *F*; tethers from PMNs treated PMNs (IL-8 red circles, PMA blue triangles, and Cytochalasin D yellow diamonds) and the linear correlation (IL-8 red dashed line $F_{th}=86$ pN and $\eta_{eff}=0.3$ pN s/µm, PMA blue dotted line $F_{th}=84$ pN and $\eta_{eff}=0.34$ pN s/µm, and Cytochalasin D yellow dot-dashed line $F_{th}=31$ pN and $\eta_{eff}=0.5$ pN s/µm) are shown in the T column; multiple tethers from normal and treated (IL-8 red triangles, PMA blue circles) PNMs, and the linear correlation for normal (black solid line $F_{th}=99\;pN$ and $\eta_{eff}=2.94$ pN s/µm) and treated (IL-8 red dashed line $F_{th}=150$ pN and $\eta_{eff}=1.17$ pN s/µm, PMA blue dot-dashed line $F_{th}=176$ pN and $\eta_{eff}=0.8$ pN s/µm) are shown in M column. **BFP** row from [49,52]. N column, tether forces pulled at different velocities (red circles 2, blue triangles 5, and yellow diamonds 15m/s); T column, Latrunculin treatment drastically reduced the force needed to pull a tether (blue triangles) at 10m/µs in comparison with normal neutrophils (red circles). Multiple tethers (M column) implied by multiple discontinuities of 109 pN attributed to single tether detachments. **OT** row from [56]. A similar behavior to what was observed with BFP for both normal and treated. **AFM** row from [83,84]. Tethers pulled with AFM showed a more abrupt transition at crossover. To date, only multiple (M column) tether pulling has been studied, showing multiple discontinuities attributable to single tether detachments (40–100 pN); single tether force jumps (shown in the table in the AFM row) of EA hy926, HB, and CHO cells were affected (30–50% reduction in jump amplitude) by various latrunculin and hyaluronidase treatments. **FC and IM** row from [7,48]. Single tether force during neutrophil rolling experiments at different wall shear stresses $\tau_{w}=6$, 8, and 10 dyn/cm${}^{2}$ before detachments, calculated with the NLD model [77]. During rolling, the jerky-tumbling motion is characterized by fluctuations in the pulling velocity, which induces fluctuation in the tether forces.

Most of the MAT experiments were technically limited by a constant pipette aspiration pressure, which implies that tethers were extracted at a constant velocity. More recent investigations have improved the setup allowing to impose a variety of pulling velocity patterns [33,101]. Another important limitation is that, although Equation (13) is derived from the solution of the Naiver–Stokes Equation [44], in reality, the sphere is attached to a forming tether. The tether is viscoelastic with a relaxation time depending on the pulling velocity ($\tau =0.3/v_{pull}^{-0.75}$[52,56,77,102]. Thus, the tether elongation rate (and as a consequence, the velocity of the attached sphere) is not constant over time, strongly limiting the method’s usability to resolve the complete viscoelastic behavior of tether extraction and instead giving information about the stationary force. Nevertheless, tethers pulled with MAT at physiological velocity ($v_{pull}$ ranging from 6 to 40 µm/s [12,65,97]) have been described with the following equation:

(14) $$F_{t}=F_{th}+2\pi \;\eta_{eff}\;v_{pull},$$

where $F_{th}$ is the threshold force required for the tether extraction to occur and $\eta_{eff}$ is the effective viscosity of the tether [31,47,50]. As shown by Figure 3, for single tether extraction, it has been estimated $F_{th}=45$ pN and $\eta_{eff}=1.8$ pN s/µm.

Due to the high surface density of leukocyte microvilli, more than one microvillus could be in contact with the endothelium at the onset of rolling and multiple tethers could be extracted, as shown by some flow chamber studies [5,11,35,46]. Using MAT, double tether extraction from neutrophils was highlighted [100]. Since the tethers are in parallel, the threshold force and effective viscosity for double tether extraction are about twice as large as those corresponding to single tether extraction (Figure 3). The presence of double tethers represents a mechanism that further stabilizes rolling because it can decrease the force on each bond much more effectively than single tethers under the same shear stress.

The effect of neutrophil activation on tether extraction was studied with MAT after neutrophil treatment with the chemokine interleukin-8 (CXCL8 or IL-8) or the protein kinase C activator phorbol 12-myristate 3-acetate (PMA). Although the authors did not provide a possible underlying molecular mechanism, both of these treatments doubled the tether pulling threshold force and decreased the effective viscosity by 80% [50] (Figure 3). To test the role of the cytoskeleton in tether formation, the authors performed these measurements on neutrophils incubated with the actin-depolymerizing agent cytochalasin D, and they found that this treatment reduced the tether pulling force by 40%. The drug disrupts the actin filaments, and as expected, the membrane flows easier into tethers [69,70,71] since cytochalasin D reduces membrane adhesion energy $W_{0}$ in Equations (6) and (8).

Girdhar and Shao [34] observed simultaneous tether extraction from ECs and PMNs, with a modified version of MAT, where an endothelial cell was used as a force probe. To show that tethers are extracted simultaneously from leukocytes and ECs when they are separated after a brief contact and adhesion, the cells were labeled with different membrane markers. Their fluorescence was observed during tether extraction and retraction, showing composite tethers. During leukocyte rolling, the pulling force due to blood flow is equally exerted on both the leukocyte and the endothelial cells. As a result, both cells’ crossover forces may be overcome, then two in-series tethers with the adhesive bond in the middle can be extracted. This is expected to reduce the force per bond even more [31], making the rolling even more efficient.

### 3.2. Tether Extraction with Biomembrane Force Probe

Both the BFP and the MAT are based on a micropipette manipulation system. The difference between these two techniques is that the BFP has a proper force transducer. In contrast, the MAT does not have a force transducer but, instead, fluid dynamic principles derived the measured force. The BFP uses a bead affixed on an aspirated red blood cell as the force transducer (see Figure 2, BFP row).

The red blood cell in the BFP serves as a spring of well-known characteristics. Tracking the RBC’s deformation through high-resolution methods of video image analysis incorporated in an optical microscope, it is possible to directly measure the force at a time resolution of 1500 frames per second. Different from the micropipette aspiration, tethers extracted with BFP show complete viscoelastic dynamics. For each pulling velocity, BFP gives measures of force vs. time rather than only a single value. In fact, from solid mechanics, it is possible to derive that, for small displacements, the RBC behaves as a Hookean spring with a spring constant $k_{RBC}$ mainly governed by the membrane tension $\sigma_{m}$ and geometrical parameters, i.e.,

(15) $$k_{RBC}≃2\pi \frac{\sigma_{m}}{\ln\left[4R_{0}^{2}/R_{p}R_{b}\right]}.$$

In Equation (15), $R_{0}$, $R_{p}$, and $R_{b}$ are the radius of the outer spherical portion of the cell, the pipette, and the adhesive contact between the glass bead and red blood cell, respectively [49]. As mentioned previously, the micropipette aspiration pressure Δp affects the membrane tension of the RBC through Equation (7), allowing experimentalists to accurately tune the stiffness of the probe over a wide range (0.2−2 pN/nm).

RBCs are usually biotinylated by covalent linking to an amine-reactive PEG-biotin polymer, saturated with streptavidin, and washed for later assembly with a biotinylated glass bead. The glass beads were coated with biotin to covalently bind them to RBCs and with P-selectin for binding to PSGL-1 on the tips of the neutrophil microvilli. Tethers extracted with BFP provided a complete description of the force vs. time behavior during tether extractions at constant pulling speed, showing that the two-phase behavior strongly depends on the pulling speed (see BFP in Figure 3). The phase after crossover followed an exponential fitting (typical of a viscoelastic response) given by

(16) $$F\left(t\right)=F_{\infty }-\left(F_{\infty }-F_{⊗}\right)exp\left[-\left(t-t_{⊗}\right)/\tau \right],$$

where $F_{\infty }$ is the fluid-like plateau force and τ is the relaxation time. Tethers exhibited a shear-thinning response where the plateau force increases weakly with the extrusion speed $v_{pull}$, i.e.,

(17) $$F_{\infty }≃60v_{pull}^{0.25}.$$

The increase in force following the increasing pulling velocity is presumably due to friction effects derived from plasma membrane intermonolayer frictions, remodeling of the cytoskeleton, and breakage of the cytoskeleton-membrane linkage, as shown by experiments performed in cells and lipidic vesicles with an actin cortex [69,90]. The pulling speed did not affect the force to extract a tether but reduced the amount of local membrane reservoir accessible by a tether. Indeed, as shown in Figure 3 (BFP row), the total tether length ($L^{*}=v_{pull}\times t^{*}$) before breakage (at the instant $t^{*}$) diminished with an increase in pulling speed (pulling at $v_{pull}=2,\;15,\;\mathrm{and}\;50\;$µm/s, the breakage lengths were $L^{*}=1.6,\;1.3,\;\mathrm{and}\;1\;$µm, respectively).

BFP measurements showed that the neutrophil incubation with the actin polymerization inhibitor latrunculin A reduced the tether pulling force by ∼60% (Figure 3, BFP row). This finding is further proof of the role of the adhesion energy of the membrane to the cytoskeleton $W_{0}$ (see Equations (6) and (8) [86,88,89]). The inhibition of F-actin formation compromises the elastic component in the tether. Both untreated and latrunculin-treated tethers failed after about 0.3s, which implies the same elongation ($L=v_{pull}\times 0.3$). This corroborates the hypothesis that tether failure may depend on the amount of membrane reservoir (excess membrane) and the local membrane accessible to each tether [69,70].

### 3.3. Tether Extraction with Optical Trap

Optical traps (OT, sometimes also referred to as laser trap or optical tweezer) have been used for pulling nanotube tethers from neutrophils [56,98,101], other cell types [57,103,104] and lipid vesicles [59,65,102,105,106] to investigate the membrane mechanical properties. The setup is similar to BFP and MAT, consisting of a single micropipette that holds the cell of interest by applying a pressure Δp. Different from the BFP, the protein-coated bead is trapped by a laser beam, which is under the objective of a microscope (Figure 2, OT row). The focal spot can trap particles in the experimental chamber and serves as a mechanical spring with an equivalent stiffens $k_{l}$, as follows:

(18) $$k_{l}=P\times \sigma_{l},$$

where *P* is the laser power (measured in *W*) and $\sigma_{l}$ is a dimensional constant (typically $\sigma_{l}≃0.07$ pN/(nm W) [61]). The stiffness of laser traps is very low, the lowest among the techniques illustrated [57], and it is usually calibrated against known viscous drag forces [103,107]. Similar to BFP, OT gives a complete viscoelastic characterization of tether pulling experiments [56,103]. In fact, the micropipette is actuated by a piezoelectric stage, which imposes controlled displacements, and measuring the deflection of the bead detected by the microscope allows us to measure the force. Tethers pulled with OT exhibit similar behaviors to what was observed by BFP (Figure 3, OT row). To date, no multiple tether experiments have been reported in the literature, presumably due to the force probe’s low stiffness that prevents imposing enough forces to pull multiple tethers. As shown by other techniques [34,49,100], in Figure 3, multiple tether extraction requires a higher amplitude of forces (at least twice as much as that for a single tether).

### 3.4. Tether Extraction with Atomic Force Microscopy

Atomic force microscopy (AFM) is a probe microscope. It is a powerful tool for single molecule investigations, plasma membrane mechanical characterization, evaluation of biological samples’ mechanical properties, and the study of intramolecular interactions [59,108,109,110,111]. It uses a sharp tip mounted at the end of a flexible cantilever. Forces acting between the surface of the sample and the probe cause deflection of the cantilever that is detected by a laser beam reflected off the back of the cantilever. The cantilever has known mechanical characteristics with a bending stiffness with an equivalent spring constant $k_{AFM}$ (as low as 10 pN/nm [57,83,84,108]). The measured force is proportional to the deflection Δu of the cantilever following Hook’s law:

(19) $$F_{AFM}=k_{AFM}\times \Delta u,$$

Although AFM offers high optical resolution (down to 1 nm), the force sensitivity is lower than BFP and OT (as low as to 5–10 pN [57]). The force sensitivity is the minimal variation in force that the probe can measure. It is limited by the cantilever’s stiffness (from one to three orders of magnitude higher than OT and BFP) and thermal fluctuations. The high optical resolution and the small dimension of the cantilever tip make it suitable for studying the lifetime of molecular bonds [112,113] and for poking adherent cells [58,114]. However, different from the other techniques presented above, the absence of a micromanipulation system makes the immobilization of suspended cells challenging. Neutrophils, while rolling, are in a suspended state and, once arrested, initiate a cascade of adhesion and transmigration events that change their biomechanical characteristics [58,83]. Thus, to investigate PMN tether formation during rolling, the cells should be kept in a non-polarized state. This was assessed by Zhang et al. [83], where individual HL-60 cells were attached to the tip of a cantilever through Concanavalin A-mediated linkages. With this approach, the AFM was used to quantify the leukocyte-endothelial adhesive interaction at the whole-cell level since the HL-60 cell served as a functionalized probe. However, this precluded the possibility of using the AFM tip to pull single tethers from localized regions of the cells’ plasma membrane. The HL-60 cells were first approached against a layer of endothelial-derived adhesion molecules (including P-, E-selectins, ICAM-1, and VCAM-1) and subsequently tested on human umbilical vein endothelial cells (HUVECs) activated by tumor necrosis factor (TNF). Since the whole cell was first approached to the adhesive layer and then retracted, the experiment illustrated multiple rupture events before final detachment. The magnitude of these rupture events calculated from the jumps visible on the AFM force vs. deflection curves (Figure 3, AFM row) were between 40 and 100 pN. The authors did not directly observe tethers due to the diffraction limitation of the microscopy. With a similar approach, Chu et al. [111] compared the adhesion of the human monocyte cell line THP-1 to immobilized Vascular Cell adhesion Molecule 1 (VCAM-1) with or without stimulation by co-immobilized monocyte chemoattractant protein (MCP-1). THP-1 cells form tethers similarly to neutrophils. THP-1 cells were immobilized onto the cantilever tip and probed onto Petri dishes functionalized with adhesion molecules. The experiments revealed that the formation of longer membrane tethers prolonged cell attachment when cells were stimulated with MCP-1. Interestingly, the mechanical work of detachment, obtained integrating the force over the retraction distance (the area under the curve), drastically increased (10 fold increase) when cells were stimulated as both the total detachment distance and the maximum force detachment increased. The increase in detachment distance was attributed to the formation of longer (2.5-fold) membrane tethers.

To directly observe tethers, a side-view AFM instrument design was introduced by Chaudhuri et al. [58]. The combination of atomic force microscopy with a side-view fluorescent imaging path enabled direct imaging of cellular deformation and cytoskeletal rearrangements along the axis of loading (Figure 2, AFM row). As mentioned, the biomechanical characteristics of adherent cells differ from non-polarized states [115]. The force–deflection curves from those experiments showed peaks of magnitudes one order of magnitude higher compared with experiments performed by Zhang et al. [83], where the cells were kept in a non-adherent non-polarized state. Another approach to clearly resolve tethers was proposed by Sun et al. [84]. They used a variety of cells, including Chinese hamster ovary (CHO) cells, a malignant human brain tumor cell line (HB), and an endothelial cell line (EA hy926), which were incubated with streptavidin-conjugated fluorescent Q-dots and attached to the cantilever. An in-house-built force measurement device using an inverted microscope, based on the design and operation of an AFM, clearly showed the formation of multiple tethers. As in the previous AFM tether experiments, the contaction–retraction experiment resulted in a series of rupture-like discontinuities in force separated by multiple plateau of forces (Figure 3, AFM row). Since the magnitudes of the discrete force steps (≃35 pN) between consecutive plateau were comparable, the authors interpreted them as the simultaneous elongation and sequential loss of multiple membrane tethers formed between the cell and the cantilever. For all cells analyzed, single tether jumps showed a marked decrease (30–50%) when cells were treated with latrunculin or hyaluronidase (degrades hyaluronan, the ligand for CD44), confirming once more that tether extraction is a complex biomechanical phenomenon depending on the coupling of membrane mechanics and membrane–cytoskeleton adhesive interaction. In particular, inhibiting F-actin actin polymerization with latrunculin seems to affect the force magnitude that a single tether can bear (table in Figure 3, AFM row) more (up to 50%) than removing the hyaluronan backbone of the glycocalyx at the membrane level (up to 35%). As shown in Figure 3, in contrast to pulling neutrophils with the OT and BFP, the impossibility to extract single tethers prevented us from clearly recognizing crossover events (from surface protrusion to tether extraction) because the crossover is hidden in the overall cell integral response where multiple tether are extracted at the same time.

### 3.5. Flow Chamber and Intravital Microscopy Experiments

Flow chamber and intravital microscopy experiments are extremely useful tools because they can either create or reproduce an in vitro or in vivo environment with fluid shear stress matching the physiological conditions observed in blood vessels [14,116,117,118,119,120]. Direct visualization of neutrophil tethers forming under venous flow conditions was demonstrated in platelets on protein-coated flow chambers at various physiological shear stresses using high-resolution differential interference contrast (DIC) video microscopy [11]. Since then, a great deal of interest has been devoted to neutrophils rolling in postcapillary venule in vivo or in vitro flow chambers over a wide range of wall shear stresses (0.5 to 50 dyn/cm${}^{2}$) [6,7,35,45,46].

The introduction of quantitative dynamic footprinting (qDF) coupled to an in vitro microfluidic flow chamber assay provided a quantitative analysis of the role of tethers in facilitating neutrophil rolling at shear stress ranging from 6 to 10 dyn/cm${}^{2}$ [7,24]. qDF is an adaptation of total internal reflection fluorescence (TIRF) microscopy and allows for the estimation of z-distances from the coverslip in rolling neutrophils’ footprints. Sundd et al. [24] showed that a neutrophil rolling on P-selectin under high shear stress forms 3–4 long tethers, extended up to 16 m behind the rolling cell. Neutrophils can form slings [7,24], cell-autonomous adhesive structures derived from detached tethers at the rear of the cell that swing over to the front, reattach to the substrate, and form new bonds with the cell, thus becoming load-bearing again. Slings stabilize rolling via two mechanisms; on slings, PSGL-1 is organized in patches that can reform molecular bonds with L-selectin that become loaded again with the cell’s rolling, and they also bind to the neutrophil cell body through the integrin LFA-1 binding to ICAM-2. Sling formation and rolling over is less common than tether formation; thus, its contribution to rolling stabilization is less compared to tethers [7,48]. qDF provides important quantitative information on the morphology of the neutrophil footprint, rolling velocity, length of tethers and slings, and tether elongation rate. These parameters allowed us to calculate the tether force’s time course while the neutrophils rolled (Figure 3, FC and IM row). In fact, feeding the instantaneous tether elongation rate to the NLDs model (Equations (10) and (11) [77]), the force fluctuation can be calculated as other tethers bear load and detach. Most tethers break in the range between 40 and 90pN. The elongation rate geometrically depends on the rolling velocity, and thus, the jerky-tumbling motion characterized by fluctuations in the rolling velocity induces fluctuation in tether forces. When one of the tethers breaks, there is a sudden increase in rolling velocity and the loss in bearing force has to be rebalanced on the remaining tethers. As shown in Figure 3 (IM and FC row), in contrast, the rolling tether endures forces higher than the value at breakage. This suggests that breakage does not depend only on the value of the force but is a more complex mechanism than a simple thresholding force. Thorough investigations are needed to further investigate the biomechanical molecular mechanism behind tether breakage, accounting for membrane stresses, local amount of membrane reservoir, adhesion energy, and friction effects. Imaging tethers and slings in vivo is challenging because they are short-lived structures with a diameter (110–200 nm) below the Abbe-resolution limit of light microscopy. With qDF in vitro, the tether anchoring points appear as bright dots (as they are very close to the coverslip) up to 16 µm behind the rolling cell, suggesting that tethers can be at least that long. In the same way, slings (up to 22 µm long) appeared in front of rolling neutrophils. qDF microscopy imaging only records the first ∼100 nm and does not allow for observing the tether-to-sling transition directly. Marki et al. [5,35,46] introduced a versatile method to demarcate the plasma membrane of neutrophils by a Ly6G-AF647 fluorescent antibody. The method was used to clearly show tether formation and breakage during rolling in vitro and in vivo, providing better quantitative information in tether dimension, morphology, and direct observation of the tether-to-sling transition, showing that 15% of tethers form slings. The mechanical role of tethers during rolling was demonstrated once more by showing that, when individual tethers break, rolling neutrophils significantly accelerate immediately (jump ∼2 µm [5,46]), proving that the tether was bearing a significant amount of load. Slower-rolling neutrophils formed tethers that detached at the tether anchor point (where the tether is attached to the endothelial substrate). However, some of the tether-forming neutrophils rolled at a faster rate, and the tethers broke along their length, sometimes multiple times, forming detached tubular particles called ENDS [35]. At ∼10 dyn/cm${}^{2}$ WSS, about 6% of rolling mouse neutrophils formed copious numbers of ENDS. Interestingly, these structures were long and elongated immediately after formation but contracted and rounded up over the next 4 h. ENDS are 100−fold elevated in blood plasma of septic patients [35].

## 4. Insights from Tethers Pulled from Lipid Membranes Vesicles

The plasma membrane has a very complex composition and dynamic organization that has motivated the development of various simpler models serving as approximations for the biomechanical properties of cellular membranes [61,121]. Giant unilamellar vesicles (GUVs) are spherical liposomes composed of phospholipid bilayers, closely resembling the composition and structure of cells’ membranes. GUVs have been extensively investigated because they are conventionally accepted as the simplest biomimetic model to approximate properties of cellular membranes [61,121,122]. The lipid composition of vesicles can be varied, and tethers (or nanotubes) can be extracted to investigate how membrane composition affects the lipid membrane’s mechanical properties, such as bending modulus, membrane tension, and pulling force to extract tethers [65,66,68,95,122,123,124].

Cuvelier et al. [61] presented an in-depth analysis of tethers from GUVs, showing how the membrane tension affects the force and tethers’ ability to coalesce. When two tethers are sufficiently close to each other, they can merge into one single nanotube. The minimum angle between two tethers to remain separated depends on the membrane tension, bending modulus, and membrane composition [61,125]. Although tether coalescence was not observed during tangential tether extraction [98], whether it happens in rolling neutrophils is not known. Cuvelier et al. [61] demonstrated that if two nanotubes are close enough, merging is energetically the most favorable configuration because it reduces membrane tension and the use of a membrane reservoir. Coalescence in neutrophils’ tethering could be another mechanism to stabilize rolling, reducing the membrane tension and increasing the amount of membrane reservoir.

GUVs have been used to investigate how the cytoskeleton and the crosslinking proteins affect the biomechanics of tether pulling [62,63,64]. Guevorkian et al. [63] prepared liposomes containing an actin cortex to mimic the cell cortex’s behavior. The biotinylated GUVs were inserted inside a microfluidic channel and subjected to different flow regimes (between 4 and 200 µm/s), and the nanotube was pulled from a streptavidin-coated microstick attached to the liposome. The microstick was held in place while the flow exerted a tunable shear force to the GUV. The elongation of nanotubes showed a viscoelastic behavior similar to tether extraction from neutrophils, where an initial viscoelastic regime is followed by a viscous regime (so-called Marangoni flow) after crossover (as highlighted by the NLD model in Equations (10) and (11) [77]). Interestingly, at each value of the shear force, GUVs with a polymerized actin cortex showed shorter nanotubes, higher crossover force, and slower elongation rate compared with GUVs without polymerized actin. This study highlighted the role of adhesion energy $W_{0}$ from the membrane on the cytoskeleton. As shown by Equation (8), the actin cytoskeleton and the crosslinking proteins reinforce the membrane by increasing the threshold force to pull a tether.

## 5. Discussion and Conclusive Remarks

In this review, we analyzed the current understanding of the biomechanics of neutrophil tether formation. Tethers are ∼200 nm thin and ∼20 µm long nanotubes pulled from rolling neutrophils that stabilize and slow down rolling. Tethers are extracted from microvilli through a two-phase process. The crossover between microvillus extension to tether extraction occurs when the plasma membrane detaches from the underlying cytoskeleton and starts flowing around membrane integral proteins bound to the cytoskeleton into the tether. Crossover requires the pulling force to exceed a threshold. The dynamic extraction of tethers from neutrophils is a viscoelastic phenomenon in which the elastic ability to recover deformation is diminished after crossover. The NDL viscoelastic model reproduces several in vitro experiments with high precision and provides estimations of the force in tether during flow chamber experiments [7,24,48].

Several research groups have investigated the biomechanics of tether extraction experimentally with various force probe measurements. BFP and OT can characterize the viscoelastic response completely. However, these techniques use micropipette manipulation systems that alter the plasma membrane tension through hydrostatic pressure. The membrane tension plays a crucial role because the threshold force at which crossover can occur positively correlates with the membrane tension. Another critical parameter is the membrane’s adhesion energy to the cytoskeleton, which is mediated by a layer of specialized crosslinking proteins and depends on the friction between the single monolayer and on remodeling of the cytoskeleton filaments. Many experiments have shown that treatments that inhibit actin polymerization drastically reduce the force necessary to pull a tether. The inhibition of F-actin formation compromises the tether’s elastic component, reducing the adhesion energy between the PM and cytoskeleton. This aspect was studied in detail by pulling nanotubes from GUVs with an actin cortex [56]. GUVs with a polymerized actin cortex showed shorter nanotubes, higher crossover force, and slower elongation rate compared with GUVs without polymerized actin. Interestingly, nanotubes pulled from GUVs showed a two-phase viscoelastic behavior similar to tether extraction from neutrophils, where a viscous regime follows an initial viscoelastic regime after crossover.

A very recent investigation combined experiments and computer simulations [126] to study in detail the effect of linkers between membrane and cytoskeleton. The force needed to extract a nanotube was found to have a nonlinear dependence on the density of membrane–cortex attachments. Low and intermediate densities did not significantly influence the force but substantially increased for large linker concentrations. These studies are important because actin filaments have also been observed inside tethers extracted from various cells [69,70,71]. The presence of actin filaments inside neutrophils tethers has not been demonstrated yet. Still, if confirmed, it can open new horizons in the biomechanics and the molecular mechanism behind tether extraction and breakage. The presence of actin filaments within tethers inevitably reinforces the membrane, and the nonuniform distribution and crosslinking along tethers can cause the formation of weak spots where the tether could break, explaining why tethers can also break in the middle (ENDS [35]). Membrane elasticity simulations of tethers pulled quasi-statically have shown concentrations of tangential stress in the neck area, which suggests that weak spots can form there [72]. More studies combining experimental and modeling efforts are needed to highlight the molecular mechanism behind tether breakage and the formation of ENDS. ENDS are significantly elevated in the blood plasma of septic patients [35], suggesting that they could have a specific role in severe inflammations. Combinations of experiments and biomechanical modeling, including the effects of membrane tension, amount membrane reservoir, and membrane–cytoskeleton adhesive interaction, are required to gain insights into the molecular mechanism of tether formation and tether breakage.

## Data Availability

Not applicable.

## Abbreviations

The following abbreviations are used in this manuscript:

PMN	Polymorphonuclear leukocytes
EC	Endothelial cells
PSGL-1	P-selectin glycoprotein ligand
ENDS	Elongated neutrophil-derived structures
MAT	Micropipette Aspiration Technique
BFP	Biomembrane Force Probe
OT	Optical Trap
AFM	Atomic force microscopy
IM	Intravital microscopy
FC	Flow chamber
GUVs	Giant unilamellar vesicles
qDF	Quantitative dynamic footprinting
NLDs	Nonlinearly decaying spring
RBC	Red blood cell
